# Obesity, Male Reproductive Function and Bariatric Surgery

**DOI:** 10.3389/fendo.2018.00769

**Published:** 2018-12-18

**Authors:** Angelo Di Vincenzo, Luca Busetto, Roberto Vettor, Marco Rossato

**Affiliations:** Department of Medicine—DIMED, Center for the Study and Integrated Management of Obesity, Clinica Medica 3, University-Hospital of Padova, Padova, Italy

**Keywords:** obesity, testosterone, male hypogonadism, sexual function, erectile function, fertility, weight loss, bariatric surgery

## Abstract

Overweight and obesity are associated with several chronic complications, such as type 2 diabetes, arterial hypertension and atherosclerotic cardiovascular diseases, with relevant consequences for patients and public health systems. Reproductive function abnormalities, such as obesity-related secondary hypogonadism, erectile dysfunction and infertility, represent other abnormalities negatively affecting the quality of life of men suffering from obesity but, despite their high prevalence, these are often understated because of a general lack of awareness in clinical practice. Obesity and gonadal function are closely related, with obesity being associated with hypogonadism that is reversed by body weight reduction thus ameliorating reproductive and sexual health. Clinical studies specifically evaluating the impact of non-surgical weight loss on testosterone levels sometimes showed conflicting results, whereas extensive literature has demonstrated that weight loss after bariatric surgery is correlated with an increase in testosterone levels greater than that obtained with only lifestyle interventions, suggesting the role of surgery also for the treatment of hypogonadism in obese male. However, studies concerning the consequences of bariatric surgery on overall reproductive function in the male, including also sexual activity and fertility, are limited and data regarding long-term effects are lacking. Here we present a brief review summarizing the evidence regarding the interplay between obesity and reproductive abnormalities in the obese male, together with the role of bariatric surgery for the treatment of these complications, describing both the positive effects and the limitations of this procedure.

## Introduction

Appropriate management of patients with obesity needs a complete evaluation and treatment of each associated cardiovascular, metabolic and endocrine disturbance (1, 2). However, despite their high prevalence, reproductive function abnormalities are often undertreated in obese population compared to the cardio-metabolic comorbidities.

As for the other well-known complications of obesity, bariatric surgery seems to be the most effective treatment for reproductive abnormalities in obese males, frequently ensuring the complete normalization of testosterone levels, along with long-term weight maintenance, although the evidence of the effects on fertility and sexual activity is still scarce. The mechanisms linking hypothalamic-pituitary-gonadal axis, obesity and metabolic surgery are far from being completely understood, and surgical weight loss sometimes is not sufficient to ameliorate sexual function and, consequently, the quality of life. To this regard, we performed a comprehensive review of the available literature to summarize the actual evidence concerning the interplay between obesity and reproductive abnormalities and the role of bariatric surgery for the treatment of these complications, analyzing the possible underlying mechanisms but also exposing its limitations.

## Obesity and Male Reproductive Function

Obesity is associated with a constellation of endocrine disturbances and, among them, reproductive function abnormalities are now recognized as relevant clinical problems in subjects with obesity. It is well-known that in women obesity is associated with menstrual irregularity, reduced fertility, higher prevalence of polycystic ovary syndrome (PCOS) (3), but also with gestational diabetes, pregnancy-related hypertension and increased risk of obesity in the offspring. In men with obesity, hypogonadism, erectile dysfunction (ED) and reduced fertility represent complications seriously affecting patient quality of life and health (4–8). Thus, their control may represent another therapeutic target in these subjects.

### Sex Hormones in Obesity

Abnormal levels of sex hormones are often observed in men with obesity. Male-obesity secondary hypogonadism (MOSH) represents an endocrine dysfunction with a reported prevalence of about 45% in moderate-severe obesity (9). Furthermore, it has been reported that the prevalence of hypogonadism in obese male is higher in the presence of type 2 diabetes (T2D) (10, 11).

MOSH is characterized by different signs and symptoms, such as sexual dysfunction, depression, fatigue, decreased lean body mass and also reduced mineral bone density, which further impact on patients' health. Hormonal abnormalities characterizing MOSH are represented by decreased free and total testosterone plasma levels, together with decreased sex-hormone binding globulin (SHBG) and increased estradiol plasma levels. Usually, in this situation, gonadotropins are inappropriately low or normal, resembling a hypogonadotropic hypogonadism, but in a less percentage of patients with metabolic syndrome higher gonadotropin levels may be observed (12).

Low testosterone and high estradiol plasma levels are common features of the metabolic syndrome, and an inverse relationship between body mass index (BMI) or waist circumference and testosterone plasma levels over all age group of patients has been extensively shown (13–15). To this respect, it is largely accepted that the development of MOSH depends on adipose tissue expansion and adipocyte dysfunction. An elevated waist circumference is the expression of increased visceral adipose tissue (VAT), which can result in an enhancement of *in-situ* aromatase activity (16). This enzyme is highly expressed in adipose tissue and is thought to be responsible for the increased conversion of circulating testosterone to 17 β-estradiol in men with obesity, favoring the development of secondary hypogonadism. At the same time, this condition leads to a vicious cycle in which low testosterone contributes to maintaining high body weight and excessive abdominal fat deposition (17). In fact, testosterone may be responsible for several metabolic effects influencing also adipocyte biology by interacting with the androgen receptor that is expressed in adipose tissue, thus preventing visceral fat accumulation and improving insulin sensitivity. Furthermore, testosterone might exert a protective role from gluco-toxicity damage on pancreatic β-cells, due to an antioxidant activity on pancreatic islets preventing the β-cell apoptosis (18) or to a modulation of the renin-angiotensin-aldosterone pathway by reducing the expression of angiotensin II type 1 receptor (AGTR1), as previously reported (19). These effects may be lost with the reduction of circulating testosterone levels, and this may explain the relationship between low testosterone plasma levels and development of insulin resistance and T2D. This could be the case not only for men with MOSH, but also for age-related late-onset hypogonadism as well as in men undergoing androgen suppression for prostate cancer. Sex hormones balance has a crucial role in energy homeostasis, energy expenditure and then body mass composition. To this respect, it is interesting to note that the effects of testosterone on adipose tissue seem mediated also by skeletal muscle. In fact, *in vitro* experimental studies have shown that testosterone may interact with androgen-receptor to promote myogenic commitment of pluripotent mesenchymal cell and inhibit adipogenic differentiation via Wnt signaling (20, 21). Furthermore, increased serum levels of leptin and pro-inflammatory cytokines produced by inflamed adipose tissue (such as IL-6 and TNF-α) might have an additional suppressive effect on reproductive function. In particular, leptin has been hypothesized to negatively affect testicular steroidogenesis acting both at central and peripheral level, diminishing the pulse amplitude of luteinizing hormone releasing hormone (LHRH)/luteinizing hormone (LH) or through the activation of its receptor present on Leydig cell (22, 23).

It is also relevant to note that metabolic complications associated with obesity, T2D in particular, further affect gonadal function and testosterone levels. Different studies have reported that low testosterone levels predict the development of T2D in men and are inversely correlated with dyslipidemia and blood pressure levels (24–27). In agreement with this observation, it has been reported that higher testosterone levels are associated with a lower risk of T2D (28). Finally, androgen deprivation treatment in patients with prostate cancer is also related to an increased risk for the development of T2D (29). On the other hand, testosterone replacement therapy has been shown to be effective in weight loss and glucose homeostasis in patients with hypogonadism and metabolic syndrome.

Different mechanisms have been suggested to explain how insulin resistance, T2D and hypogonadism are interconnected in males. The effect of hyperinsulinemia on lowering testosterone concentrations has been evaluated many years ago (30, 31), showing that insulin acts both at central level, impairing the activity of gonadotropin releasing-hormone (GnRH) secreting neurons, and peripherally, where it may suppress SHBG synthesis (32), LH signaling (33), or modulate Leydig cell activity (34). The association becomes even more complex when associated complications develop. Obstructive sleep apnoea is a condition characterized by low circulating testosterone levels, but testosterone replacement in these patients has been associated with a worsening of clinical symptoms (35). Furthermore, it has been reported that the relationship between testosterone plasma levels and non-alcoholic fatty liver disease (NAFLD) shows a gender difference: low testosterone levels are independently associated with NAFLD in men and post-menopausal women (36), whereas testosterone levels are inversely associated with NAFLD in women (37), and increased levels of testosterone in PCOS patients are observed in association with NAFLD (38, 39).

Thus, the treatment of hypogonadism in patients with obesity, T2D or metabolic syndrome should consider also to the management of associated metabolic complications. To this respect testosterone replacement therapy in hypogonadal obese men has been previously suggested. Different studies have confirmed that testosterone replacement therapy in obese men leads not only to the amelioration of sexual activity, but also to the reduction of body weight, improved insulin sensitivity and reduction of other well-known cardiovascular risk factors, such as cholesterol plasma levels and inflammatory markers (40–43).

**Figure 1:**
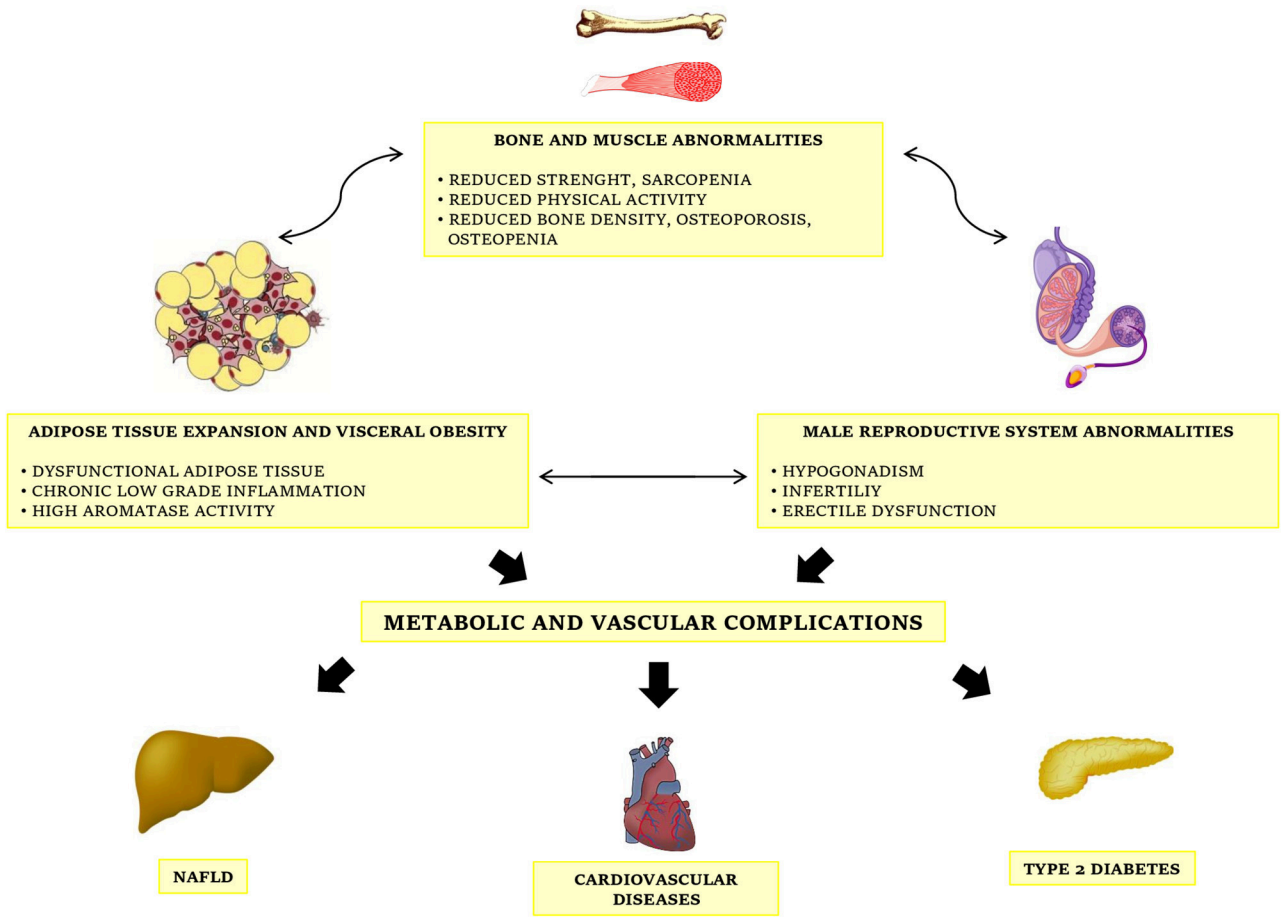
The complex interconnections between obesity, metabolic diseases and male gonadal function.

Taken together, this evidence shows a bidirectional interconnection between testosterone deficiency and metabolic diseases, a relation becoming further intriguing considering that low testosterone plasma levels have been recognized as an additional risk factor for cardiovascular disease (44, 45), and thus hypogonadism may account as another clinical complication of obesity (Figure 1). So, the identification of MOSH in obese subjects is strongly recommended for a complete risk stratification.

### Sexual Activity, Erectile Function, and Fertility in Obesity

Obesity has a relevant negative impact not only on sex hormones levels but also on the overall sexual performance. Furthermore, according to some observations, adipose tissue expansion seems to influence also gonadal activity and spermatogenesis, leading to the reduction of fertility in obese males. BMI seems to be independently correlated with erectile function, with a high prevalence of overweight and obesity in patients suffering for ED. In comparison with normal BMI subjects, patients with a BMI higher than 28,7 Kg/m^2^ show a 30% higher risk of ED (46), and it has been reported that patients with obesity waiting for bariatric surgery show a higher probability to suffer from ED with respect to those following non-surgical weight loss programs (47). In addition, in patients with ED, the presence of obesity and associated metabolic complications seems to affect further the severity of the disease, even reducing the responsiveness to phosphodiesterase 5 (PDE5) inhibitors (largely used for ED treatment) in subjects with higher BMI levels (48). Apart from the effect of MOSH and abnormal sex hormones levels, vascular and microvascular abnormalities associated with VAT expansion and chronic low-grade systemic inflammation, may represent another possible link between obesity and ED. In fact, obesity is associated with several clinical markers of subclinical atherosclerosis, such as endothelial dysfunction and impaired activity of endothelial nitric oxide, which may be at least in part responsible for the development of ED in this population. With these patho-physiological mechanisms in mind, the overlap between obesity and ED seems to represent a point of connection between adipose tissue expansion and vascular complications. Then, the presence of ED in obese subjects may account for an increased cardiovascular risk.

On the contrary, conflicting results have been observed in studies evaluating the correlation between BMI and sperm parameters, such as sperm concentration and total sperm count, although it is generally accepted that men with obesity seem to have a higher risk of azoospermia or oligozoospermia (49–51). The mechanisms by which obesity may influence fertility are complex and heterogeneous. Hyperinsulinemia and hyperleptinemia, classical features of obesity, may have a direct effect on spermatogenesis because of oxidative stress and inflammatory state, with loss of sperm DNA integrity (52, 53). Obesity might act centrally as for hypogonadism, influencing the hypothalamus-pituitary-testis axis. Furthermore, it is also possible that increased VAT may be responsible for an increased scrotal temperature which could impair testicular function.

The different treatments of obesity, such as lifestyle modification, pharmacological and surgical therapy are associated with an improvement of the different obesity-associated diseases along with weight loss, and body weight reduction appears to be effective also for MOSH. The different studies on the effects of behavioral modifications on male obese reproductive function have shown discordant results (54–56). On the contrary, a huge amount of literature has demonstrated that surgical weight loss is associated with an increase in testosterone plasma levels higher than that obtained with lifestyle modifications alone, probably reflecting the correlation between the severity of obesity and the grade of gonadal activity impairment. In fact, after surgical weight loss, an increase of testosterone plasma levels is observed in patients with MOSH, with a magnitude of androgen rise greater in those patients losing more weight (57), often leading to completely revert hypogonadism (58). However, at the moment, the effects of bariatric surgery on reproductive function in obese males remain not entirely known, and their explanation might be relevant to define future therapeutic options in obese males with MOSH and/or other reproductive abnormalities with contraindication to bariatric surgery.

## Role of Surgical Weight Loss on Reproductive Function in Male Obesity

Considering their prevalence, obesity-associated gonadal dysfunctions may represent an important target for bariatric surgery. A recent meta-analysis has shown that, in patients undergoing bariatric surgery, PCOS was present in 36% of women whether MOSH was present in 64% of men, with a high percentage of remission after surgery (96% for PCOS, 87% for MOSH) (59). Below we present a brief description of the effects of bariatric surgery on male sex hormones plasma levels, ED and semen quality.

### Bariatric Surgery and Sex Hormones Plasma Levels

As previously mentioned, exogenous testosterone has been proven to be effective in hypogonadal obese men also in the amelioration of anthropometric and metabolic parameters (40–43, 60), and it could be a helpful treatment strategy in this population. However, androgen replacement therapy still has a reduced diffusion among physicians, probably because of apprehension for the possible adverse effects, such as exacerbation of OSA, worsening in lower urinary tract symptoms, and the risk of neoplastic prostate disease. In addition, exogenous testosterone may exert a negative feedback on releasing of gonadotropins which may further influence the fertility status. Weight loss has a great role in increasing testosterone levels, and a rapid weight loss obtained with lifestyle modifications has shown a beneficial effect in subjects with obesity. Niskanen et al. demonstrated that after a 9-week very-low-calorie diet there was an increase in SHBG and total testosterone plasma levels that were maintained also during weight control period (61). The results of a recent study evaluating the effects of medical treatment (dietary intervention and physical exercise) with the aim of 10% weight loss, confirmed a significant increase of total testosterone and a reduction of 17-β estradiol plasma levels after weight loss (62). Other studies evaluating the effects of calorie restriction on testosterone plasma levels reported conflicting results, while more promising results have been obtained from studies associating diet and testosterone replacement therapy (63).

On the other hand bariatric surgery is linked to greater effects on reproductive hormones abnormality in the male. Extensive literature has shown the positive effect of bariatric surgery on MOSH and this effect is irrespective of the type of surgery. Calderón et al demonstrated that significant postoperative outcomes on weight loss, HOMA index, fasting glucose, waist circumference, but also total and free testosterone plasma levels were not different between different bariatric surgery procedures such as gastric bypass, adjustable gastric banding or sleeve gastrectomy (64, 65). A limitation of these studies is represented by the heterogeneity of surgical procedures, sometimes considering the effects both of restrictive and malabsorptive surgery, which might be responsible for a relevant bias. However, after surgical weight reduction, many different studies have shown that the increase in total testosterone plasma levels is directly associated with amelioration of metabolic parameters whatever the surgical technique utilized, such as biliopancreatic diversion, vertical banded gastroplasty, and Roux-en-Y gastric by-pass (RYGB) (66–68), confirming the existence of an intricate hypothalamic-gonadal-adipocyte axis.

A positive effect of bariatric surgery on sex hormones plasma levels is commonly observed after a mid- or long-term follow up, but it can also present a rapid onset, already at 1 month from sleeve gastrectomy or RYGB (69, 70). With the maintenance of weight loss and appropriate weight control, the effects of bariatric surgery on sex hormones levels may be observed also for longer times. Pham et al recently performed an ancillary analysis from STAMPEDE trial, evaluating free and total testosterone levels 5 years after sleeve gastrectomy or RYGB in obese men with T2D. Patients who underwent bariatric surgery showed a higher increase in total and free testosterone levels with respect to medical patients in the long period (84.1 vs. 9.6%, *p* = 0.008, and 47.4 vs. −2.2%, *p* = 0.013), without significant changes in estradiol levels (71). Interestingly the increase in free testosterone observed in this study was associated with a reduction in body weight, high sensitivity-C reactive protein and leptin plasma levels, but not with an improvement in glycemic control. These results may be explained by the development of more severe abnormalities of gonadal function occurring when obesity coexists with other metabolic diseases, showing that adipose tissue dysfunction and systemic inflammation further influence gonadal activity in addition to insulin resistance and glucose metabolism alterations.

To this regard, the effects of bariatric surgery on gonadal function may be directly linked to body weight loss and, in particular, to visceral fat mass reduction. A recent study evaluating the correlation between sex hormones plasma levels and VAT (measured with magnetic resonance imaging), confirmed that visceral fat area is the main parameter associated with testosterone levels at baseline and, in addition, showed that after bariatric surgery visceral fat area is negatively associated with the increase in total testosterone plasma levels (72). VAT is well known to represent the most relevant risk factor associated with cardio-metabolic risk in patients with obesity Furthermore it probably accounts also for the development of hypogonadism in obese male, since abnormalities of adipocyte function in VAT result in negative effects on endocrine system, given that the imbalanced production of adipokines and inflammatory mediators such as leptin, IL-6, and TNF may directly influence testicular activity *in vitro* (23, 73), but evidence is lacking *in vivo*. Moreover, the reduction in VAT, that expresses higher aromatase activity with respect to the subcutaneous adipose tissue (SAT), may result in a reduced conversion of circulating testosterone leading to the reduction of estradiol and increase in testosterone plasma levels.

However, there are other clinical circumstances which may influence the amplitude of the effects of metabolic surgery on sex hormones recovery beyond weight loss *per se*. Age represents a relevant determinant for amelioration of MOSH, probably because younger subjects could have a greater response of testicular function to hypothalamic-pituitary hormones (in particular to the increase of LH levels) (74) or conversely because of an abnormal hypothalamic responsiveness to circulating sex hormones (75). To this respect, another limitation of the studies evaluating the post-surgical recovery of hypogonadism is represented by the age of studied subjects, who are usually young men when at the time of bariatric surgery.

The improvement of testosterone plasma levels may favor a condition in which the restoration of muscle strength and resistance leads to increased physical activity, amelioration of obesity-related sarcopenia and positive effects also on osteopenia and articular function, globally contributing to weight loss maintenance (76). Considering the experimental evidence regarding the influence of bone on testicular function (77), metabolic surgery may present these intriguing additional effects. To this regard, Samavat et al observed that osteocalcin, a bone hormone produced by osteoblasts, which seems to play a role on the regulation of testis function, increased after bariatric surgery, and this increase was parallel with the rising in free testosterone levels (78).

### Bariatric Surgery and Erectile Function

A healthy lifestyle can preserve or restore erectile function in men, in particular in men with obesity who are at higher risk for ED because of associated metabolic and neuro-vascular abnormalities. Esposito et al showed the beneficial effects of lifestyle changes (reduced caloric intake and increased physical activity) on ED in subjects with obesity in a randomized controlled trial in which the intervention group, receiving detailed advice about how to achieve a loss of 10% or more by, obtained a significant improvement in the International Index of Erectile Function (IIEF) score (79). The beneficial effects of a moderate (10%) weight loss on sexual function have been confirmed by others (80).

As demonstrated by a recent meta-analysis, bariatric surgery leads to a significant improvement of ED as assessed by IIEF (81). ED is commonly reported by patients with severe obesity in the pre-operative period, but it may be improved by surgical induced weight loss. Dallal et al. showed that gastric bypass may revert also a severe sexual dysfunction (evaluated in this study with the Brief Male Sexual Function Inventory), with the excess of weight loss independently predicting the entity of the improvement (82). As for the increase in total testosterone plasma levels, bariatric surgery seems to guarantee an early amelioration of erectile function at six but also 1 month from surgery (83–85).

Surgical weight loss can ameliorate erectile dysfunction, as evaluated by IIEF questionnaire, probably through a modification in sex hormones plasma levels, in particular increasing follicle-stimulating hormone (FSH), total and free testosterone (86). Nonetheless it is also possible that bariatric surgery acts with a more complex mechanism. In a retrospective study conducted on Chinese obese patients with ED, Kun et al demonstrated the beneficial effects of RYGB on erectile function and showed the correlation between IIEF score and arterial integrity (both cavernosal and carotid artery, evaluated with Doppler ultrasound), irrespective of baseline BMI (87). In fact, the amplitude of the improvement of sexual function on IIEF seems to be independent of the increase of sex hormones plasma levels, confirming a more profound alteration of sexual function in obese patients which is not associated with testosterone activity alone. In a prospective observational case-series study, Mora et al observed, using a multivariate regression analysis, that variations of BMI and not hormonal and metabolic factors were independent predictors of IIEF score improvement at 1 year from bariatric surgery (88).

However, there are no univocal data about long term effects of surgical weight loss, and bariatric surgery does not always guarantee a complete normalization of erectile function. This observation is probably linked to the weight regain or to the persistence of metabolic abnormalities (89, 90), or also to psychological issues. A retrospective study by Ranasinghe et al evaluating a male obese population undergoing laparoscopic gastric banding showed an improvement of IIEF score without overall sexual function improvement: in particular, erectile index and orgasmic function worsened when adjusted for time (91). On the contrary, other studies evaluating pre- and post-surgical sexuality in male obese subjects, described positive effects of surgical weight loss, pointing also to the role of psychological issues on overall sexual activity (92, 93).

### Bariatric Surgery and Semen Quality

Beyond the sex hormonal status and related clinical symptoms, bariatric surgery may potentially be associated not only with the improvement of reproductive hormones and related clinical symptoms but also with amelioration of semen parameters possibly resulting in improved fertility. Samavat et al analyzed the effects of RYGB on semen quality after 6 months from the procedure in a cohort study. They observed an increased sperm count, motility and ejaculate volume after treatment although not reaching the statistical significance. In addition, they also showed a reduction in sperm DNA fragmentation and semen IL-8, unconventional parameters of semen quality (94). Increase in total testosterone plasma levels may be partially responsible for these modifications, but these results also confirm the hypothesis regarding a deep, complex effect of obesity on reproduction, testicular activity and fertility in men, which may be reversed only partially by weight loss. Obesity may affect directly the biochemical activity of the testis, as demonstrated by lower levels of inhibin B observed in obese men, and bariatric surgery can potentially improve also this parameter (95). On the contrary, a retrospective study by Legro et al showed no amelioration in semen parameters of obese men that underwent gastric bypass (96). A previous prospective randomized study of Reis et al comparing surgical procedure and medical follow-up showed similar results: gastric by-pass obtained a significant increase in IIEF score, total and free testosterone levels in the intervention group, but did not affect sperm quality (97). In a prospective study of El Bardisi et al evaluating effects of sleeve gastrectomy on semen parameters, semen quality was not affected by surgical weight loss except for subgroups of men with pre-existing azoospermia or oligospermia (98). Some reports have also described the possibility that bariatric surgery may negatively influence sperm parameters, probably because of nutritional deficiency and induction of a systemic catabolic state with consequent accumulation of toxic metabolites (99–101). However, taken together, these data are relatively too weak to definitely clarify the situation, and more controlled studies in large populations are needed regarding this topic.

## Conclusion

Bariatric surgery seems to be effective to improve free and total testosterone plasma levels in obese men, even if more evidence is needed about changes of other hormones such as gonadotropins and adrenal sex steroids. In addition, while testosterone plasma levels improvement is maintained for a long-term, restored erectile function seems not to be a durable effect, even if increased semen quality is not guaranteed after metabolic surgery. These conditions may be explained by the complex pathophysiology of reproductive function abnormalities occurring in men with obesity. In particular, adipose tissue expansion and consequent biochemical activity impairment (cytokines and adipokines production, increase peripheral androgens aromatization) may be responsible for reduced levels of testosterone while different and even more complex mechanisms, involving not only sex hormones levels but also neuro-vascular abnormalities and gonadal specific alterations, could be responsible for erectile dysfunction and infertility. To this regard, metabolic surgery may present some limitations; however, with respect to non-surgical weight loss, bariatric surgery remains the most effective treatment for a rapid improvement of global sexual activity and hypogonadism, so it could be considered as a relevant option for severely obese hypogonadal males.

## Author Contributions

DVA and RM equally wrote the article. VR and BL revised the paper.

### Conflict of Interest Statement

The authors declare that the research was conducted in the absence of any commercial or financial relationships that could be construed as a potential conflict of interest.
